# Triglycerides and total cholesterol concentrations in association with IFG/IGT in Chinese adults in Qingdao, China

**DOI:** 10.1186/s12889-018-5286-z

**Published:** 2018-04-03

**Authors:** Jing Cui, Jianping Sun, Wei Wang, Nafeesa Yasmeen, Ma Ke, Hualei Xin, Qing Qiao, Aiguo Ma, Zulqarnain Baloch

**Affiliations:** 1 School of Public Health of Qingdao University, No. 38 Dengzhou Road, Qingdao, 266021 China; 2 Qingdao Municipal Center for Disease Control and Prevention, Qingdao, 266033 China; 3 Qingdao Institute of Preventive Medicine, Qingdao, 266033 China; 4 College of Traditional Chinese Medicine, Shandong University of Traditional Chinese Medicine, Jinan, 250355 China; 5 Institute of Microbiology, University of Agriculture, Faisalabad, Pakistan; 6 Key Laboratory of Food Safety Risk Assessment, Ministry of Health, China National Center for food safety Risk Assessment, Beijing, 100021 China; 7 Department of Public Health, University of Helsinki, Helsinki, Finland; 8 College of Veterinary Medicine, South China Agricultural University, Guangzhou, 510642 China

**Keywords:** Triglycerides, Total cholesterol, Adult onset IFG/IGT

## Abstract

**Background:**

To investigative the association of triglycerides (TG) and total cholesterol (TC) concentrations with impaired fasting glucose/ impaired glucose tolerance (IFG/IGT) in Chinese adults.

**Methods:**

The population-based cross-sectional diabetes survey was conducted in 2006 and 2009 in Qingdao, separately. 4400 participants (1 793 men and 2607 women) were include in current analysis. IFG/IGT was defined according to fasting plasma glucose (FPG) and/or 2 h post-load plasma glucose (2 h PG). Logistic regression models and areas under receiver operating characteristic curves (AUROC) were performed to estimate the associations between TG, TC levels and IFG/IGT.

**Results:**

Spearman analysis showed that serum TG and TC was independently and positively associated with FPG and 2 h PG. As compared with normoglycaemia, the odds ratio[(95% confidence intervals), OR(95%CI)] for IFG/IGT corresponding to hypertriglyceridemia (HTG) were 1.61 (1. 17, 2. 22) in men and 1.57(1.15, 2.14) in women for TG and accompany with Hypercholesterolemia (HTC) 1.56 (1.15, 2.13) and 1. 20 (0.93, 1.54) for TC, when adjusting for confounding factor. The AUROCs of TG, TC for IFG/IGT were relatively smaller (0.50 < AUROC< 0. 7) in both gender. The optimal cut-offs for TG and TC was 1.61, 4.91 in men and 1. 24, 5. 32 in women, respectively.

**Conclusions:**

Evaluated TG in both gender and TC in men were independently associated with the present of the IFG/IGT, yet, could not be an authentic predictors of IFG/IGT in both men and women in current Chinese population.

## Background

Pre-diabetes refers to a condition in elevating plasm glucose, and that is consider normal levels but is not high enough to be classified as diabetes. Pre-diabetes has been defined by impaired fasting glucose (IFG) and/or impaired glucose tolerance (IGT) according to the 2006 World Health Organization (WHO)/International Diabetes Federation (IDF) criteria [1]. Individuals with IFG/IGT were at about 5–10% high risk of developing diabetes worldwide [2, 3]. In China, the prevalence of IFG/IGT is surprisingly high and continues to increase. In a cross-sectional study reported in 2007–2008 involving a nationally representative sample of 46,239 adults, 20 years of age or older, the prevalence of IFG/IGT15.5% [4], and which is far higher than previously reported figures [5]. In additional, with the high epidemiological status of IFG/IGT, they also have a high risk of dyslipidemia [6].

Dyslipidemia can be defined by abnormal level of triglycerides (TG) and total cholesterol (TC). However, the association of TG, TC and IFG/IGT is still not clear. Existing researches demonstrated that hyperglycaemiawas in concert with the potential risk factors of cardiovascular diseases [7–9], for instance elevated TG [10] and TC [11]. Previously cohort study indicated that elevated TG was a high risk for diabetes in adults [12, 13]. Akehi et al. [14] also found the strong association between TC and the plasma glucose levels at 60 min. Chakarova et al. [15] examined associations of serum lipid with IFG/IGT in 1240 adults in the population-based cross-sectional study, the results showed that IFG presents with significant differences in the levels of TG and TC are significantly higher compared to the group with normoglycaemia, while TG of IGT group are increased as compared to normoglycaemia, and the differences in TC between IGT and normoglycaemia are not statistically significant.

There are some studies about the association of TG, TC with pathoglycemia in China. Previously two cross-sectional studies [16, 17] examined that elevated TG and TC are risk factors for diabetes in Chinese adults. Multivariate logisticregression analyses of a cross-sectional study involving 4 583Chinese adults reportedhigher TG was therisk factors for developing pre-diabetes [18]. However, so we have known, the association between TG, TC and IFG/IGT is still not fully elucidated at present. Therefore, we designed this study to assess the association between TG, TC and IFG/IGT in general population based on a large population-based cross-sectional survey in Qingdao, China.

## Methods

### Study population

The current study utilized data form Qingdao Diabetes Prevention Program (QDDPP). The general individuals aged 35–74 years who had lived in Qingdao city for at least 5 years were valid in 2006 and 2009, respectively.This study applied strict exclusion criteria: 1) previously diagnosed diabetes; 2) fasting plasma glucose (FPG) and 2 h post-load plasma glucose (2 h PG) based newly diagnosed diabetes; 3) missing for glycated haemoglobin (HbA1c), TG, TC, waist circumference (WC), body mass index (BMI).

### Setting

This study was conducted in three rural districts (Jiaonan, Jimo and Huangdao) and three urban districts (Sifang, Shibei, and Shinan) and in 2006 and 2009. All collected data from six districts were combined to QDDPP, andinputted by uniform staff in the study.

### Sampling methods and size

**Fig. 1:**
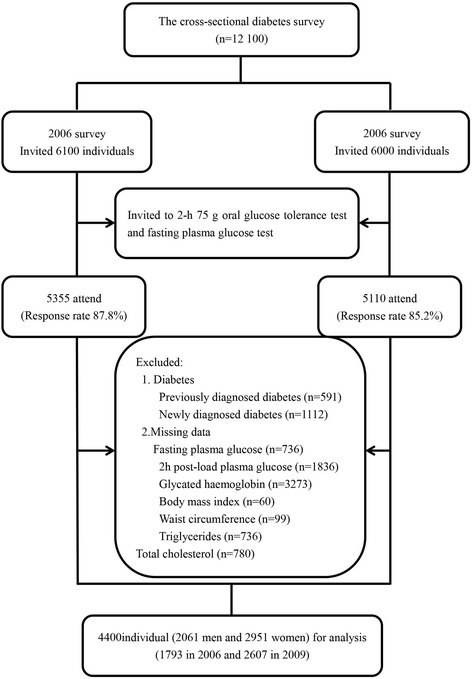
Flow chart of the Participants included in the cross sectional analysis

The QDDPP survey included both stratified random samples. The data included 10 465 individuals (5355 in 2006 and 5110 in 2009) of 12,100 citizens (6100 in 2006 and 6000 in 2009) with a response rate of 87. 8% in 2006 and 85.2% in 2009, respectively (Fig. 1). Following the exclusion criteria, 4400 participants (1793 men and 2607women) were included in this current analysis.

### Ethical considerations

All the participants voluntarily signed the informed consent before their participation, and the security, anonymity and privacy of participants was respected rigorously in this study. The ethics committee of the Qingdao Municipal Center for Disease Control and Prevention formally reviewed and approved our study in 2008 and 2014, and the ethical approval number was Document No. 1 and Document No. 7, respectively.

### Data collection

The standardized questionnaire was used in face-to-face interview by trained doctors and nurses. Age (years), gender (men/women), marital status, smoking status, alcohol-drinking status, educational attainment, personal monthly income, family history of diabetes were collected through questionnaire. The criteria of marital status were recorded into unmarried or married. The criteria for smoking habit and alcohol-drinking habit were the following: 1) non-smokers or current smokers, 2) non-drinkers or current drinkers. The criteria for educational attainment were defined into school years ≤9y or > 9y. The criteria of personal socio-economic clusters were categorized into personal monthly income≤599 Chinese Yuan (CNY), 600–1999 CNY, or ≥ 2000 CNY. Family history of diabetes was defined; at least one family member diagnosed with diabetes (including parents, sibling and/or off-springs, etc). Height and weight of the participants were measured in only light clothes and barefoot by trained doctors and nurses, and BMI was computed as weight (kilograms) dividing by height squared m^2^). WC was measured to the nearest 0. 1 cm at the narrowest point between the rib cage and the iliac crest. Blood pressure of participants was measured on the upper right arm in siting position.

After an overnight fast of at least 10 hours, blood samples were collected from the antecubital vein into a vacuum tube containing sodium fluoride, and transported in a dark box with ice to the laboratory and stored at − 80 °C freezer within no more than 6 hours. A standard 2-h 75 g oral glucose tolerance tests (OGTT) was performed in all subjectswith no history of diabetes. All detection index of blood samples were analyzed in the central laboratory of Qingdao Endocrinology and Diabetes Hospital: 1) FPG; 2) 2 h PG; 3) fasting serum TG; 4) fasting serum TC; 5) Serum alanine aminotransferase (ALT) 6) serum gamma-glutamyltransferase (GGT)

### Variable definitions

Categories of glucose tolerance were defined according to the 2006 World Health Organization (WHO)/International Diabetes Federation (IDF) criteria [1]. Newly diagnosed diabetes was defined as FPG ≥ 7.0 mmol/l and/or 2 h PG ≥ 11. 1 mmol/l.IFG/IGT was defined as IFG (FPG 6. 1–6. 9 mmol/l), and/or IGT (2 h PG 7. 8–11.0 mmol/l). Normoglycaemia was defined as FPG < 6. 1 mmol/l and 2 h PG < 7. 8 mmol/l. Participants with previously diagnosed diabetes and/or newly diagnosed diabetes were excluded from the current analysis.

TG and TC were categorized according to the most recent guideline from Chinese Heart Association [19]. Participants were divided into three TG categorical groups as follows: normal TG (TG < 150 mg/dl), borderline high TG (150 ≤ TG < 200 mg/dl), hypertriglyceridemia (HTG) (TG ≥ 200 mg/dl). Participants were also subdivided as follows: normal TC (TC < 200 mg/dl), borderline high TC (200 ≤ TC < 240 mg/dl), and hypercholesterolemia (HTC) (TC ≥ 240 mg/dl).

### Statistical analysis

Continuous variables and categorical variables are presented as means [95% confidence intervals (95% CI)] and number (percentage).The general linear model for continuous variable and the Chi-squared test for categorical variable were used to compare the difference between different glucose categories. Correlations between TG, TC and plasma glucose were assessed by spearman correlation coefficient. Logistic regression analysis was used to estimate the odd ratio (OR) and 95% CI for the association of serum TG, TC with IFG/IGT in men and women. Area under receiver operating characteristic (AUROC) was investigate the association of TG, TC and IFG/IGT. According to AUROC, the diagnostic values of TG, TC were assessed: an AUROC of ≤0.5 was considered a chance result; 0.5–0. 7, low accuracy; 0. 7–0. 9, moderate accuracy;≥0. 9, high accuracy. The optimal cut-offs for TG and TC was selected based on the sensitivity and specificity. Statistical analyses were performed using IBM SPSS Statistics 18.0. A *P* value less than 0.05 was considered to be statistically significant.

## Results

In this study, 35.4% (634/1793) men and 35.0% (912/2607) had IFG/IGT. The baseline characteristics of the study population were presented in Table 1. Compared with normal glycaemia, those individuals with IFG/IGT were older, more obese, and have higher levels of systolic blood pressure (SBP), ALT, GGT, TG and TC. Family history of diabetes, unban living and higher income level in men and less school years, lower income level and higher level diastolic blood pressure (DBP) in women with pre-diabetes was more common than in those with normoglycaemia.

**Table 1:** Baseline characteristics of participants according to glucose (*N* = 4400)

	Men		Women	
	Normoglycaemia	IFG/IGT	Normoglycaemia	IFG/IGT
Number (%)	1159 (64.6)	634 (35.4)^ǂ^	1695 (65.0)	912 (35.0)^ǂ^
Age (years)	49. 1 (48.4, 49.7)	52.5 (51.7, 53.4)^ǂ^	48. 1 (47.6, 48.5)	52.7 (52.0, 53.3)^ǂ^
Urban living	353 (30.5)	234 (36.9)^*^	766 (45.2)	405 (44.4)
Married, n (%)	1104 (96.8)	602 (95.6)	1609 (95.7)	839 (93.6)^*^
School years > 9 (%)	427 (37.0)	246 (38.9)	629 (37.2)	207 (22.8)^ǂ^
Current Smoking (yes, %)	611 (52.9)	313 (49.5)	19 (1.1)	11 (1.2)
Alcohol-drinking (yes, %)	503 (43.5)	285 (45.1)	27 (1.6)	13 (1.4)
Family history of diabetes (yes, %)	144 (13.3)	101 (16.9)^*^	301 (18.7)	172 (20.0)
Income (CNY/month), n (%)				
≤ 599	413 (36.8)	182 (29.4)^*^	720 (44.2)	468 (53.2)^ǂ^
600–1999	527 (47.0)	320 (51.8)	803 (49.3)	376 (42.7)
≥ 2000	181 (16.1)	116 (18.8)	105 (6.4)	36 (4.1)
Body mass index (kg/m2)	24.9 (24.7, 25.0)	25.7 (25.5, 26.0)^ǂ^	25.0 (24.8, 25.1)	26.1 (25.8, 26.3)^ǂ^
Waist circumference (cm)	85.2 (84.7, 85.8)	88.1 (87.3, 88.9)^ǂ^	81.1 (80.7, 81.5)	83.0 (82.4, 83.6)^ǂ^
Systolic blood pressure (mmHg)	131.8 (130.8, 132.9)	134.9 (133.5, 136.3)^*^	127.5 (126.6, 128.4)	135.3 (134.0, 136.6)^ǂ^
Diastolic blood pressure (mmHg)	85.7 (85.1, 86.4)	86.9 (85.9, 87.8)	81.9 (81.3, 82.4)	85.1 (84.4, 85.9)^ǂ^
Fasting plasma glucose (mmol/L)	5.20 (5.16, 5.24)	5.98 (5.93, 6.02)^ǂ^	5.24 (5.21, 5.26)	5.85 (5.81, 5.89)^ǂ^
2 h post-load plasma glucose (mmol/L)	5.69 (5.61, 5.77)	7.67 (7.56, 7.78)^ǂ^	6.02 (5.96, 6.08)	8.02 (7.95, 8.10)^ǂ^
Glycated haemoglobin (%)	4.34 (4.29, 4.39)	4.45 (4.39, 4.52)*	4.33 (4.29, 4.38)	4.39 (4.33, 4.45)
Alanine amino transferase (U/L)	15.6 (14.9, 16.4)	19.6 (18.5, 20.6)^ǂ^	13.6 (13.1, 14.2)	15.4 (14.7, 16.3)^ǂ^
Gamma-glutamyl transferase (U/L)	32.9 (30.0, 35.7)	43.3 (39.4, 47.2)^ǂ^	17.5 (16.6, 18.3)	20.1 (19.0, 21.3)^ǂ^
Triglycerides (mmol/L)	1.31 (1.1, 26.37)	1.59 (1.52, 1.67)^ǂ^	1. 22 (1.1, 18.26)	1.41 (1.36, 1.46)^ǂ^
Normal TG	924 (79.7)	441 (69.6)^ǂ^	1421 (83.8)	649 (71.2)^ǂ^
Borderline high TG	123 (10.6)	86 (13.6)	169 (10.0)	157 (17.2)
Hypertriglyceridemia	112 (9.7)	107 (16.9)	105 (6.2)	106 (11.6)
Total cholesterol (mmol/L)	5.14 (5.08, 5.19)	5.37 (5.29, 5.45)^ǂ^	5.22 (5.18, 5.27)	5.33 (5.27, 5.39)^*^
Normal TC	647 (55.8)	278 (43.8)^ǂ^	906 (53.5)	382 (41.9)^ǂ^
Borderline high TC	373 (32.2)	246 (38.8)	539 (31.8)	336 (36.8)
Hypercholesterolemia	139 (12.0)	110 (17.4)	250 (14.7)	194 (17.0)

Data are age-adjusted mean (95% confidence interval) or n (%) as indicated. ^*^P < 0.05, ^ǂ^< 0.001, normoglycaemia versus pre-diabetes within the same gender

**Fig. 2:**
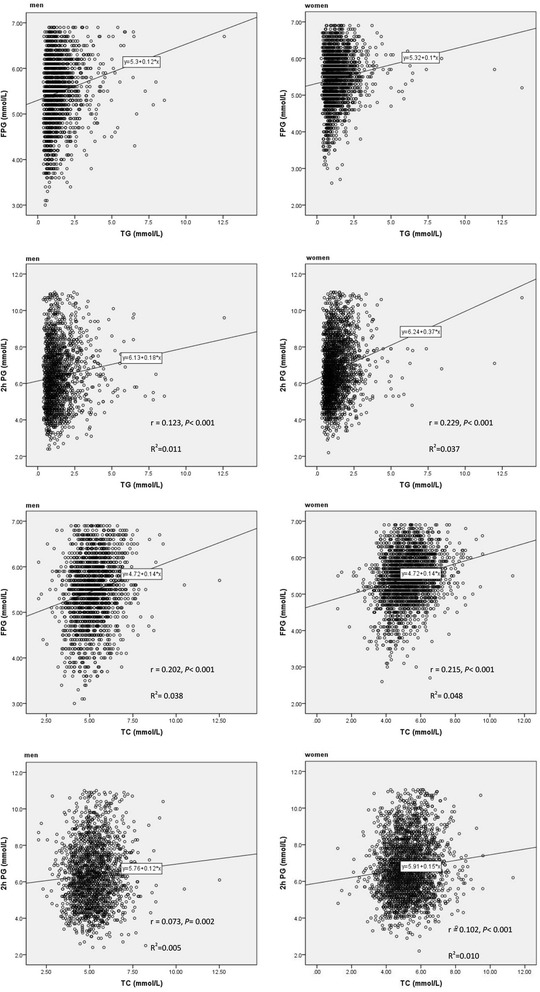
Spearman correlation between triglycerides, total cholesterol and plasma glucose

In both gender, the level of serum TG was independently and positively associated with FPG and 2 h PG (*P* < 0.05), and the similar association was between serum TC and FPG, 2 h PG (*P* < 0.05). The spearman association was shown in Fig. 2.

Table 2 showed OR for IFG/IGT in association to triglycerides and total cholesterol concentration by univariate logistic regression. In both gender, age, BMI, WC, SBP, DBP, ALT and GGT were significantly associated with a higher risk for IFG/IGT (*P* < 0.05). Unban living and higher income level were significantly associated with a higher risk for IFG/IGT in men(*P* < 0.05), while married, more school years and higher income level were significantly associated with a lower risk for IFG/IGT in men(P < 0.05). Borderline high TG, HTG and borderline high TC, HTC had a higher OR for IFG/IGT in both men and women (*P* < 0.05).

**Table 2:** Odds ratio (95% confidence interval) for IFG/IGT in association to triglycerides and total cholesterol concentration by univariate logistic regression

	Men		Women	
	OR	*P* value	OR	*P* value
Age (years)	**1.03 (1.02, 1.04)**	<0.001	**1.05 (1.04, 1.06)**	<0.001
Urban living	**1.34 (1.09, 1.64)**	0.005	0.97 (0.82, 1.14)	0.701
Married, n (%)	0.70 (0.42, 1.16)	0.167	**0.67 (0.47, 0.95)**	0.026
School years >9 (%)	1.09 (0.89, 1.33)	0.423	**0.50 (0.41, 0.60)**	<0.001
Current Smoking (yes, %)	0.87 (0.72, 1.06)	0.172	1.08 (0.51, 2.27)	0.847
Alcohol-drinking (yes, %)	1.07 (0.88, 1.30)	0.519	0.89 (0.46, 1.74)	0.742
Family history of diabetes (yes, %)	1.32 (1.00, 1.74)	0.048	1.08 (0.88, 1.33)	0.457
Income (CNY/month), n (%)		0.007		<0.001
≤599	1.00		1.00	
600–1999	**1.38 (1.10, 1.72)**	0.005	**0.72 (0.61, 0.85)**	<0.001
≥2000	**1.45 (1.09, 1.95)**	0.012	**0.53 (0.36, 0.78)**	0.002
Body mass index (kg/m2)	**1.07 (1.04, 1.10)**	<0.001	**1.11 (1.08, 1.13)**	<0.001
Waist circumference (cm)	**1.03 (1.02, 1.04)**	<0.001	**1.04 (1.03, 1.05)**	<0.001
Systolic blood pressure (mmHg)	**1.01 (1.01, 1.02)**	<0.001	**1.03 (1.02, 1.03)**	<0.001
Diastolic blood pressure (mmHg)	**1.01 (1.00, 1.02)**	0.041	**1.03 (1.02, 1.04)**	<0.001
Alanine amino transferase (U/L)	**1.02 (1.01, 1.03)**	<0.001	**1.02 (1.01, 1.03)**	<0.001
Gamma-glutamyltransferase (U/L)	**1.00 (1.00, 1.01)**	0.001	**1.02 (1.01, 1.02)**	<0.001
Triglycerides (mmol/L)		<0.001		<0.001
Normal TG	1.00		1.00	
Borderline high TG	**1.47 (1.09, 1.97)**	0.012	**2.03 (1.61, 2.58)**	<0.001
Hypertriglyceridemia	**2.00 (1.50, 2.67)**	<0.001	**2.21 (1.66, 2.94)**	<0.001
Total cholesterol (mmol/L)		<0.001		<0.001
Normal TC	1.00			
Borderline high TC	**1.54 (1.24, 1.90)**	<0.001	**1.48 (1.23, 1.77)**	<0.001
Hypercholesterolemia	**1.84 (1.38, 2.45)**	<0.001	**1.84 (1.47, 2.30)**	<0.001

Bold type indicated statistically significant values

Table 3 showed the multivariate adjusted OR between TG, TC and IFG/IGT employing backward method. The multivariate adjusted OR of having IFG/IGT was significantly higher in men with HTG,borderline high TC, HTC, and in women with borderline high TG, HTGthan in those with the normal TG, TC. The association between borderline high TG in men, borderline high TC, HTC in women and IFG/IGT was no significant, but increased risk.Concomitantly, age, SBP, ALT were significant positive association with an increased risk of IFG/IGT in both gender in multivariate logistic model. While, a significant positive association wasdiscovered between IFG/IGT and higher income level, WC in men and urban living in women as well as a significant invert association with more school years, middle income level in women. The results of the multivariable logistic analysis were not changed substantively when BMI taken a place of WC was entered into the models.

**Table 3:** Odds ratio (95% confidence interval) for IFG/IGT in association to triglycerides and total cholesterol concentration by multivariable logistic regression

	Men		Women	
TG				
Normal TG	1.00		1.00	
Borderline high TG	1. 28(0.93, 1.77)		1.67(1. 28,2. 16)*	
Hypertriglyceridemia	1.61(1.17, 2.22)*		1.57(1.15, 2.14)*	
TC				
Normal TC		1.00		1.00
Borderline high TC		1. 31(1.04, 1.64)*		1. 17(0.96, 1.43)
Hypercholesterolemia		1.56(1.15, 2.13)*		1.2(0.93, 1.54)
Age (years)	1.04(1.03, 1.05)*	1.04(1.03, 1.05)*	1.02(1.01, 1.03)*	1.02(1.01, 1.03)*
Urban living			1. 29(1.04, 1.59)*	1. 26(1.02, 1.56)*
School years > 9 (%)			0.65(0.52, 0.82)*	0.66(0.53, 0.83)*
Income (CNY/month), n (%)				
≤599	1.00	1.00	1.00	1.00
600–1999	1.52(1.1, 20.94)*	1.52(1.1, 19.93)*	0.73(0.60, 0.89)*	0.73(0.60, 0.89)*
≥2000	1.58(1.15, 2.17)*	1.60 (1.16, 2.2)*	0.67(0.43, 1.05)	0.71(0.45, 1. 10)
Waist circumference (cm)	1.02(1.01, 1.03)*	1.02(1.01, 1.03)*		1.01(1.00, 1.02)
Systolic blood pressure (mmHg)	1.01(1.00, 1.01)*	1.01(1.00, 1.01)*	1.02(1.01, 1.02)*	1.02(1.01, 1.02)*
Alanine amino transferase (U/L)	1.02(1.01, 1.03)*	1.02(1.01, 1.03)*	1.01(1.00, 1.02)*	1.01(1.00, 1.02)*
Gamma-glutamyl transferase (U/L)		1.00 (1.00, 1.00)		

The logistic regression models were employed backward. All variables of table were listed in the final models.TG category and TC category entered models separately. * *P* < 0.05 for factors associated with depression

**Fig. 3:**
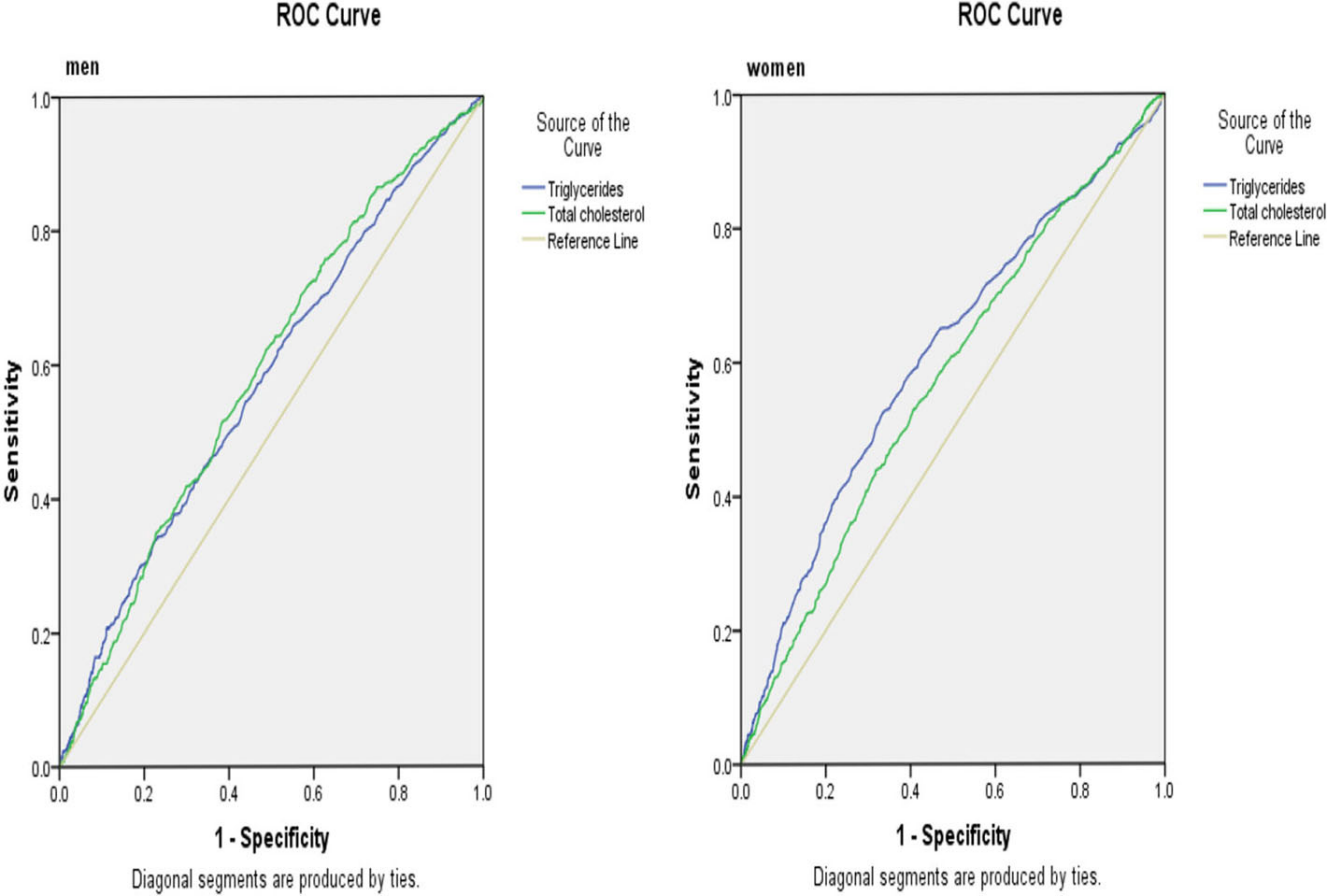
Area under the receiver operating characteristics curves of triglycerides and total cholesterol for IFG/IGT

The AUROCs of TG, TC for IFG/IGT were relatively smaller (0.50 < AUROC< 0. 7) in both gender. The optimal cut-offs for TG and TC were 1.61 (sensitivity: 0. 34, specificity: 0.77), 4.91 (sensitivity: 0.70, specificity: 0.43) in men and 1. 24 (sensitivity: 0.52, specificity: 0.67), 5. 32 (sensitivity: 0.53, specificity: 0.59) in women, respectively. The AUROCs of TG, TC for pre-diabetes was presented in Table 4 and Fig. 3.

**Table 4:** Area under the receiver operating characteristics curves of TG, TC for IFG/IGT

	AUROC (95%CI)	*P* value
Men		
TG	0.58 (0.55, 0.60)	< 0.001
TC	0.59 (0.56, 0.62)	< 0.001
Women		
TG	0.61 (0.59, 0.63)	< 0.001
TC	0.57 (0.55, 0.60)	< 0.001

## Discussion

In the current population-based cross-sectional study, spearman analysis revealed that serum TG and TC were independently and positively associated with FPG, 2 h-PG in both men and women. Multivariate logistic analysis showed HTG in both gender,borderline high TG in women, and borderline high TC, HTC in men were positively associated with IFG/IGT independent of other known risk factors. However, AUROC results showed TG and TC as predictor of IFG/IGT was low accuracy.

To the best of our knowledge, serum TG was associated with glucose. Akehi et al. [14] demonstrated that the strong association between TG and plasma glucose levels at 60 min, which hinted TG could be as a predictor for futurediabetes in young males. The cross-sectional and cohort studies of adults all indicated that elevated TG level was a positively associated with diabetic subjects after adjusted for confounding factor [13, 16, 17]. Furthermore, Rasmussen et al. reported that hypertriacylglycerolaemia was significant predictor of progression in IFG/IGT subjects, and decrease of hypertriacylglycerolaemia markedly reduced the risk of diabetes in these high-risk subjects [20]. These findings also agree with our results. In our present multivariable analysis, the associations between TG and IFG/IGT remained constantly considering the confounding factors. Furthermore, previous research demonstrated that evaluated fasting TG could predict future diabetes [21, 22]. However, TG predicting IFG/IGT TG was low accuracy, and the first in Chinese population. The previously follow-up study indicated evaluated TG increase the risk of evaluated glucose [13]. Meanwhile, TG level changes were associated with initiation of changes in lifestyle parameters [23]. The patho-physiological mechanisms of abnormal TC and glucose are intricate and hard to fully understand. TG is responsible for the bidirectional transference of adipose fat and blood glucose from the liver. Previously reported research has confirmed that excessive TG are restructured into adipose fat in liver and stored into hepatocytes [24]. While, superfluous adipose fat can reduce response to insulin signaling [25].

Yet, less research on TC and IFG/IGT has been done at present. Akehi et al. [14] found that TC was significantly associated with plasma glucose levels at 60 min and 120 min in young males. Previously cross-sectional studies [16, 17] revealed that elevated TC increased the risk for diabetes in multivariable logistic models. Meanwhile, the population-based studies [26, 27] reported that TC was a significant association with pre-diabetes after adjusting for multiple covariates. Our present analysiswassimilar but not identical to previous researches. Evaluated TC was significant positive associationin men and insignificant positive association in women with IFG/IGT, yet, couldn’t predict IFG/IGT accurately. Published study has indicated that excess TC may alter the potassium channels of beta-cells, which reduce the activity of glucokinase, and then affect insulin signaling reducing the metabolism of glucose [28].

The present study demonstrated that IFG/IGT was significant positively associated with age, urban living, WC and SBP, which were similar to published studies [18, 29, 30]. Abnormal glucose, which brings on diabetic microangiopathy, is more likely to occur as a consequence of ageing, elevated WC, and urbanization. Microangiopathy causes other series of physiological dysfunction, such as hypertension. Of note, the inverse association between and abnormal glucose had been revealed in previous and present studies [31, 32]. The individual with higher educational attainment are more interested in health. Nevertheless, the effect of personal monthly income on glucose is the controversial issues in present study, and previously studies also showeda mixed opinion on income [33, 34]. Perhaps,men with higher income have greater living and mental pressure and unhealthy lifestyle. However, women with higher income pay more attention to health in China.So, educational initiatives for individuals with abnormal glucose are necessary by present results.

As far as we know, this is the first study to investigate the association between TG, TC level and IFG/IGT in China. In addition, the present study has some strengths. Firstly, this is the relatively large number of subjects included in this population-based study to provide the association between TG, TC and IFG/IGT with high statistical power for data analyses. Secondly, all IFG/IGT subjects were defined according to FPG and/or 2 h PG, which was accepted standard criteria in current. Thirdly, we have fully captured multiple statistical models to investigate the strength of the association between TG, TC level and IFG/IGT in different populations and different types of variables. However, our study suffered from a few potential weaknesses that merit comment. The current study was from two cross-sectional studies, it is enormous challenges to evaluate the association between TG, TC and IFG/IGT. Secondly, only PFC, 2 h PG, TG and TC were performed once in the current study.

## Conclusion

Evaluated TG and TC has emphasized significantly positively associated with IFG/IGT. HTG, borderline high TC, HTC in men and borderline high TG, HTG in women had anextremelyadverse impacton IFG/IGT, however, TG and TC could not be anauthenticpredictors of IFG/IGT in this Chinese population. Additionally, further research about the association between TC, TG concentration and hyperglycemia should be conduct large-scale, multi-population-based study to confirm the conclusion in Chinese population.

## Abbreviations

2 h PG: 2-h plasma glucose
ALT: Alanine amino transferase
AUROC: Area under receiver operating characteristic
BMI: Body mass index
CI: Confidence intervals
DBP: Diastolic blood pressure
FPG: Fasting plasma glucose
GGT: Gamma-glutamyltransferase
HbA1c: Glycatedhaemoglobin
HTC: Hypercholesterolemia
HTG: Hypertriglyceridemia
IDF: International Diabetes Federation
IFCC: International Federation of Clinical Chemistry
IFG: Impaired fasting glucose
IGT: Impaired glucose tolerance
OGTT: 2 h 75 g oral glucose tolerance tests
OR: Odds ratio
SBP: Systolic blood pressure
TC: Total Cholesterol
TG: Triglycerides
WC: waist circumference
WHO: World Health Organization
